# Supplementary material for: Addressing MRSA infection and antibacterial resistance with peptoid polymers
Source: Nat Commun. 2021 Oct 8;12:5898. doi: 10.1038/s41467-021-26221-y (PMC8501045; doi:10.1038/s41467-021-26221-y)
Supplement: Supplementary file 1 — Supplementary Information [file 41467_2021_26221_MOESM1_ESM.pdf]

Supplemental Information

Addressing MRSA Infection and Antibacterial Resistance with Peptoid Polymers

Jiayang Xie,[¶1] Min Zhou,[¶1] Yuxin Qian,[2] Zihao Cong,[2] Sheng Chen,[2] Wenjing Zhang,[2] Weinan Jiang,[2] Chengzhi Dai,[2] Ning Shao,[2] Zhemin Ji,[2] Jingcheng Zou,[2] Ximian Xiao,[2] Longqiang Liu,[2] Minzhang Chen,[2] Jin Li,[3] and Runhui Liu[*,1,2]

[1]State Key Laboratory of Bioreactor Engineering, School of Materials Science and Engineering, East China University of Science and Technology, Shanghai 200237, China

[2]Key Laboratory for Ultrafine Materials of Ministry of Education, Frontiers Science Center for Materiobiology and Dynamic Chemistry, Research Center for Biomedical Materials of Ministry of Education, East China University of Science and Technology, Shanghai 200237, China

[3]Shanghai Key Laboratory of Orbital Diseases and Ocular Oncology, Department of Ophthalmology, Ninth People's Hospital, Shanghai Jiao Tong University School of Medicine, Shanghai 200011, China

Correspondence should be addressed to R.L. (rliu@ecust.edu.cn)

[¶]J.X. and M.Z have equally contributed to the manuscript.

Materials

Ethyl bromoacetate, 2-aminoethanethiol and 4-Bromo-1,8-naphthalic anhydride were purchased from Meryer Technologies in China. 4-Phenylbenzylamine, n-butylamine and n-hexylamine were purchased from Shanghai Aladdin Bio-Chem Technology Co., Ltd. Ethyl acetate (EtOAc), dichloromethane (CH_2Cl_2), tetrahydrofuran (THF), petroleum ether (PE) and triethylamine were purchased from Shanghai Titan Technology Co., Ltd. All other reagents and solvents were purchased from Shanghai Adamas Reagent and used without further purification. Synthesized intermediates were purified using a SepaBean machine equipped with Sepaflash columns produced by Santai Technologies Inc. in China.

Synthesis of monomer N$^{\beta}$-Cbz-aminoethyl-NNCA

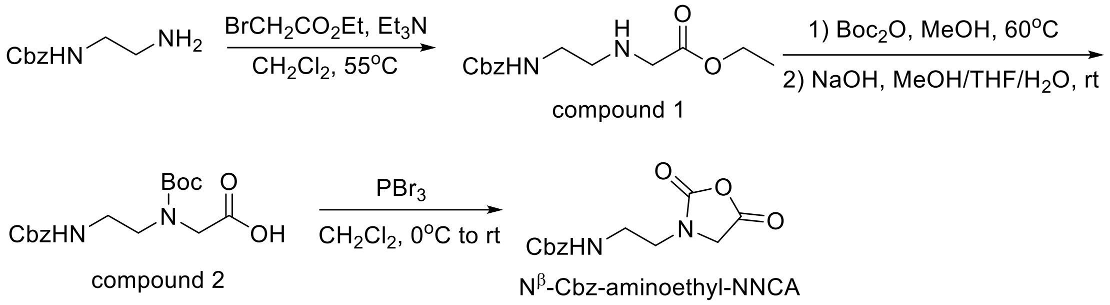

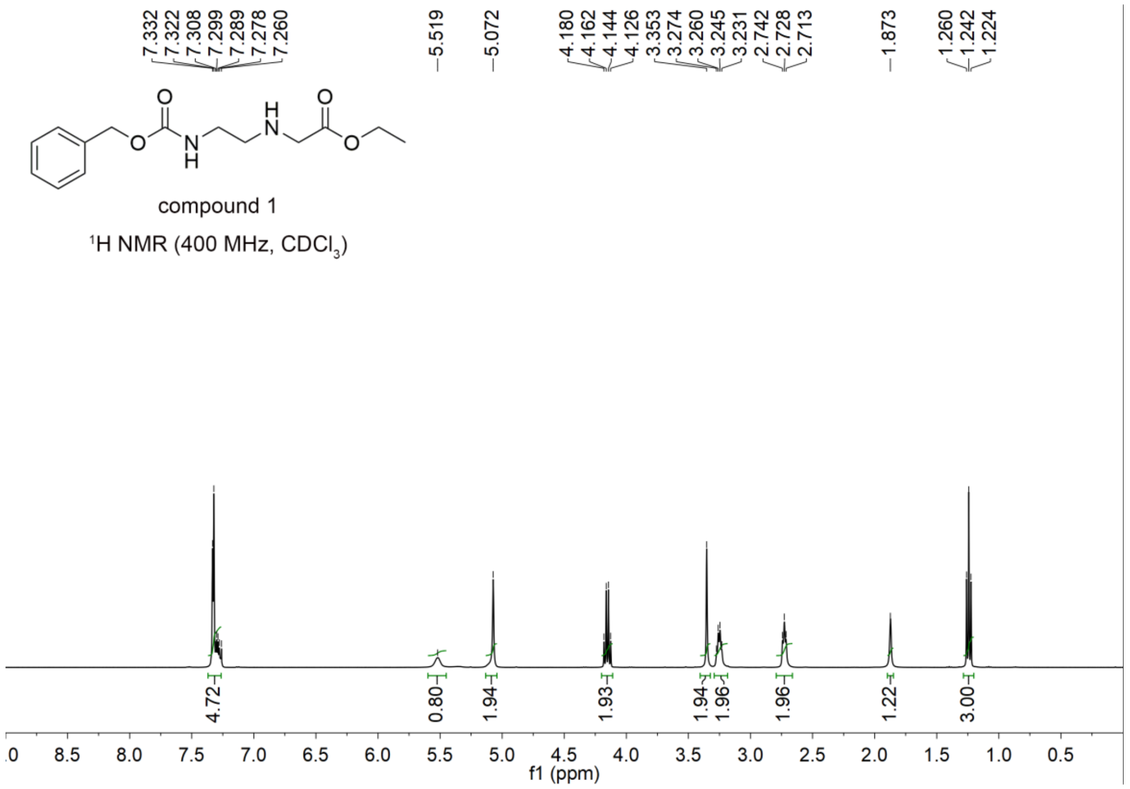

Supplementary Figure 1. ^{1}H NMR spectrum of compound 1.

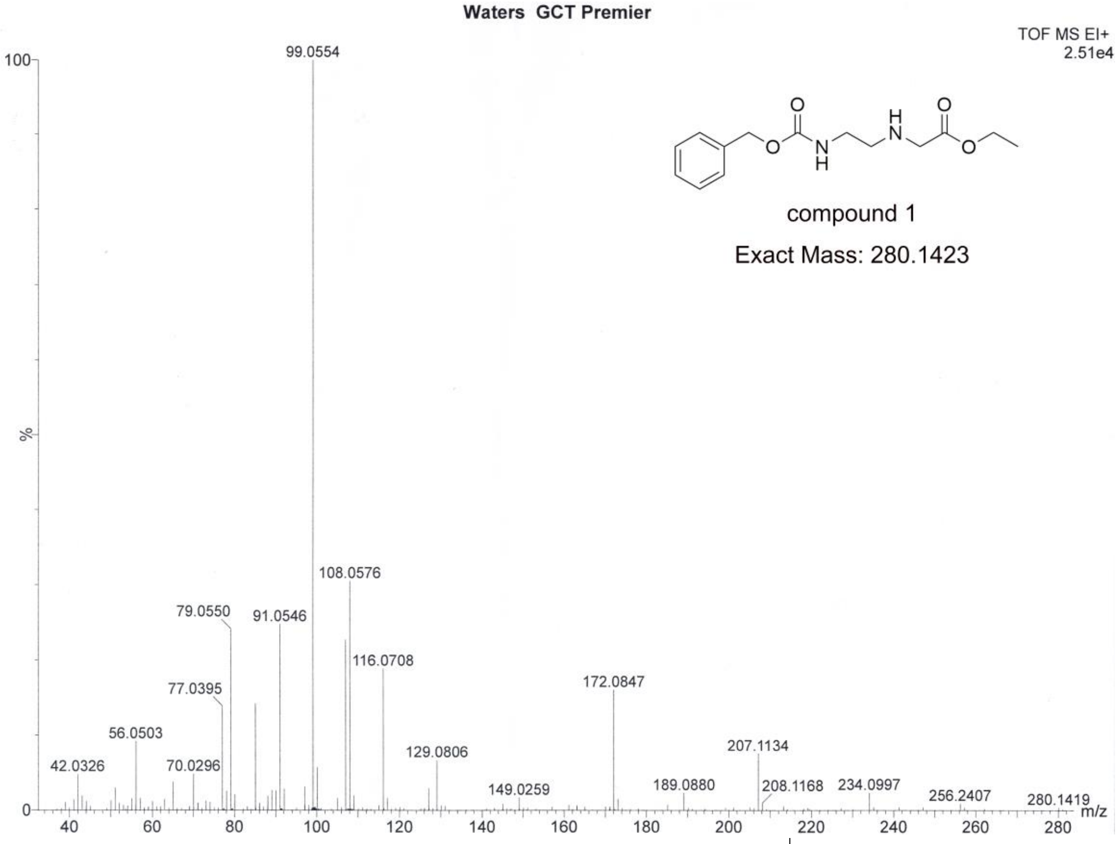

Supplementary Figure 2. HREI-MS spectrum of compound 1.

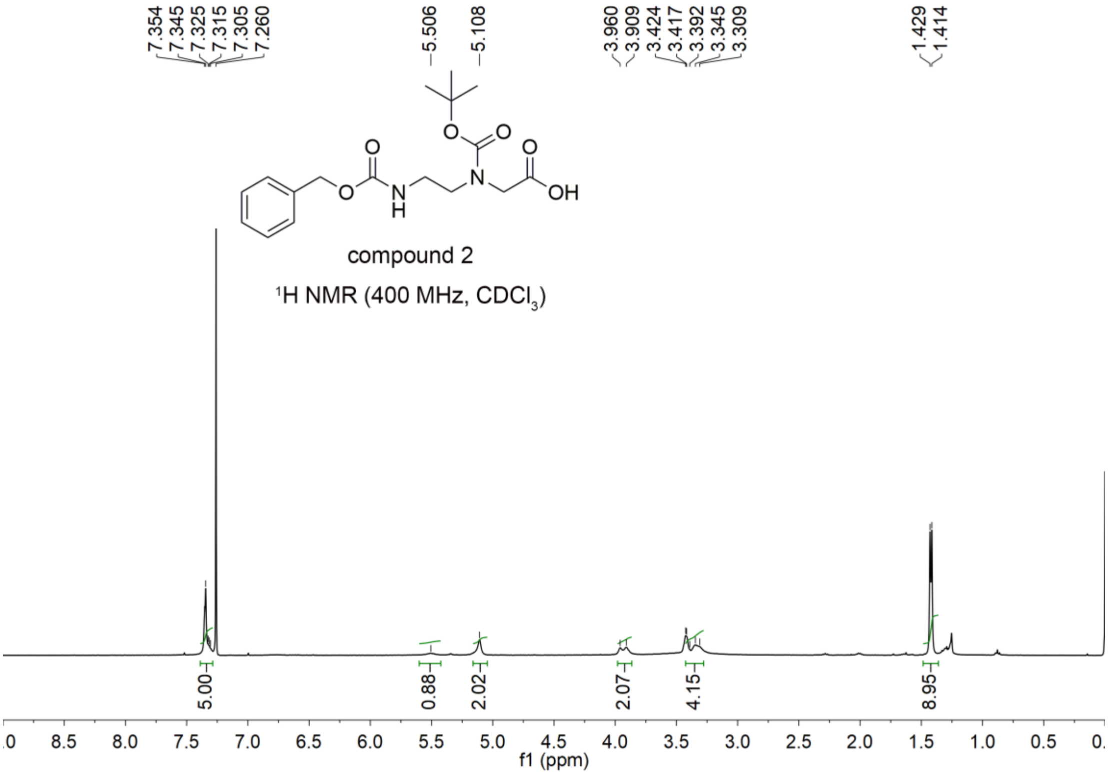

Supplementary Figure 3. ^{1}H NMR spectrum of compound 2.

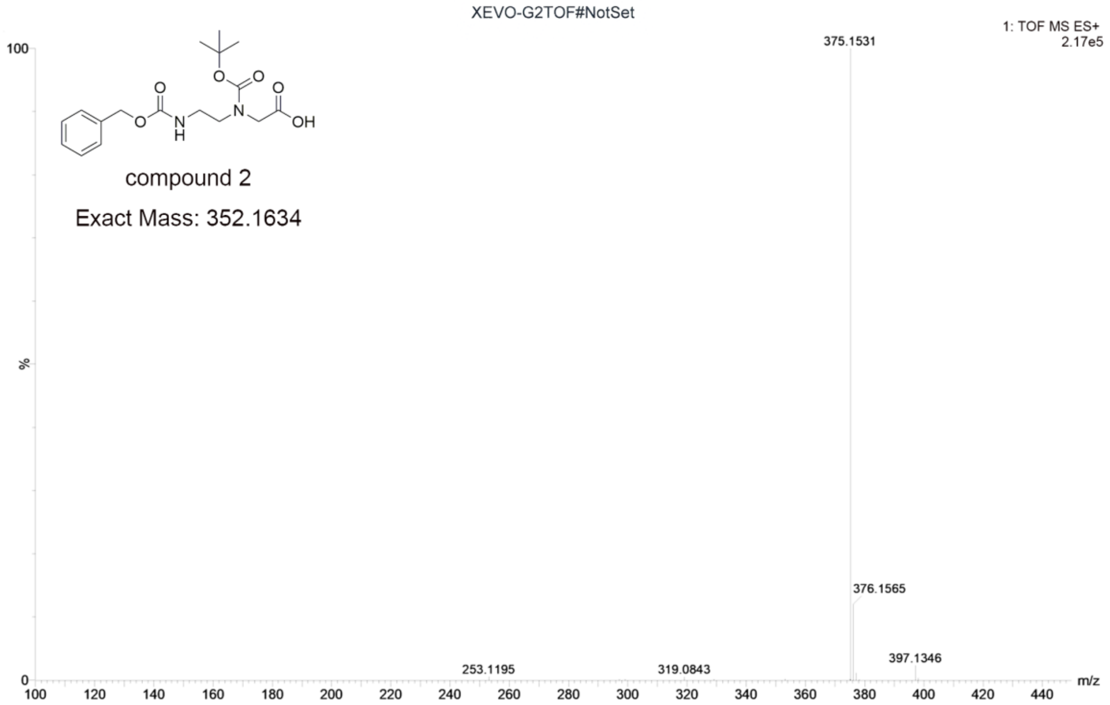

Supplementary Figure 4. HRESI-MS spectrum of compound 2.

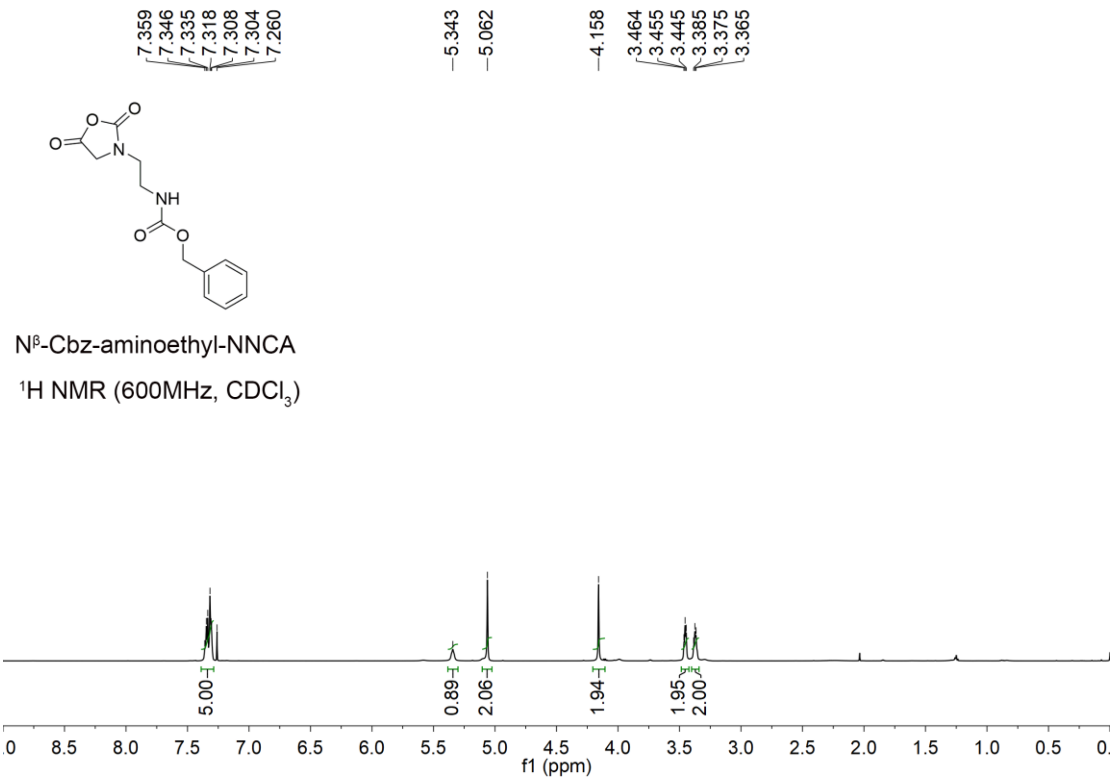

Supplementary Figure 5. ^{1}H NMR spectrum of N$^{\beta}$-Cbz-aminoethyl-NNCA.

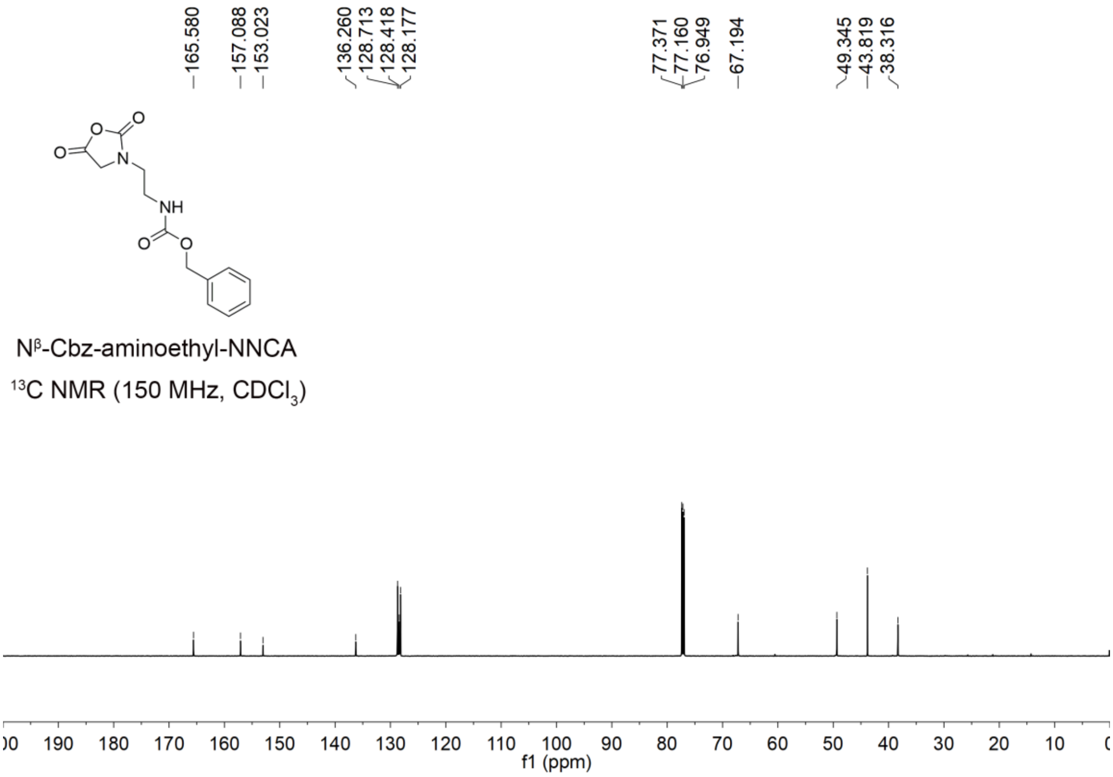

Supplementary Figure 6. ^{13}C NMR spectrum of N$^{\beta}$-Cbz-aminoethyl-NNCA.

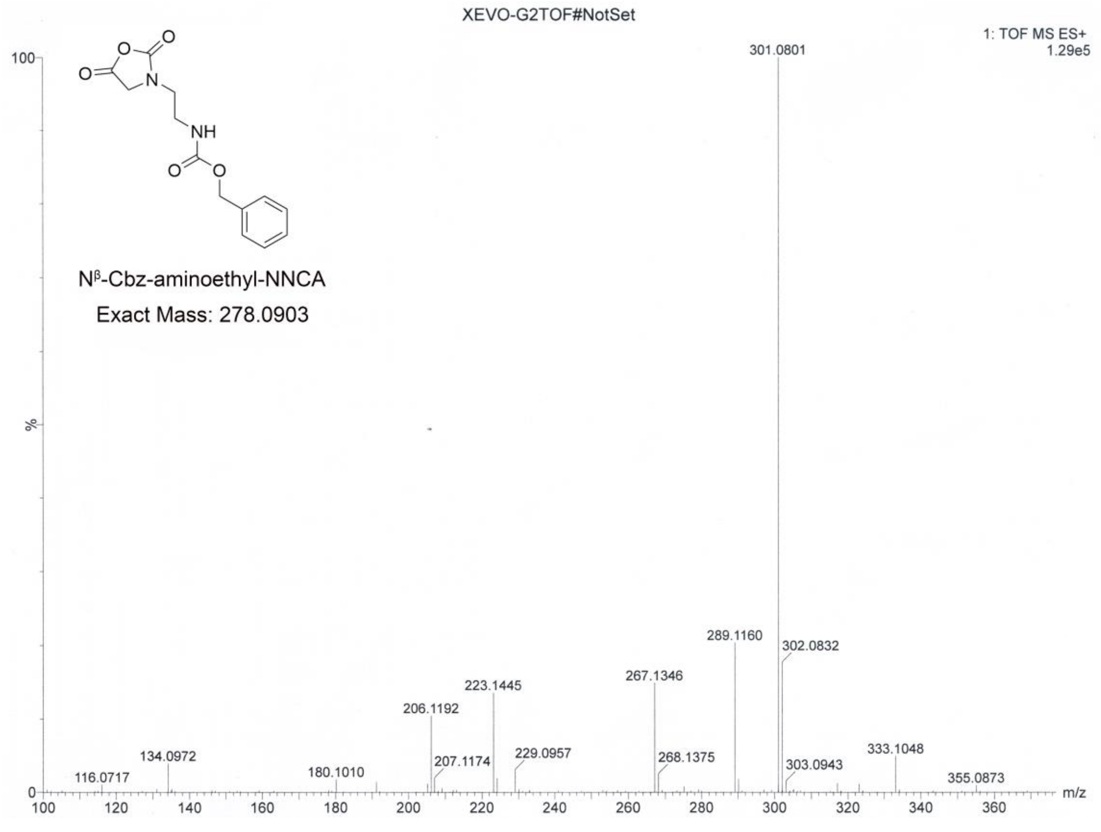

Supplementary Figure 7. HRESI-MS spectrum of N$^{\beta}$-Cbz-aminoethyl-NNCA.

Polymerization of N$^{\beta}$-Cbz-aminoethyl-NNCA.

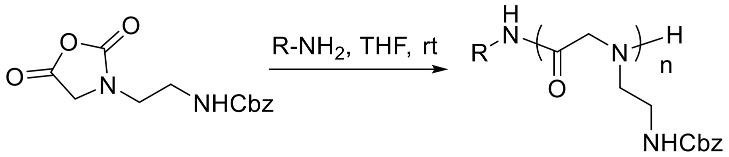

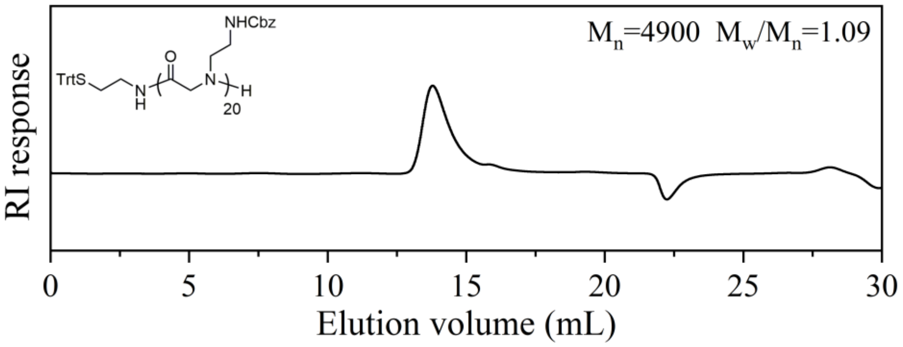

Supplementary Figure 8. GPC trace of N-Cbz protected Polymer 1 using DMF as the mobile phase at a flow rate of 1 mL/min.

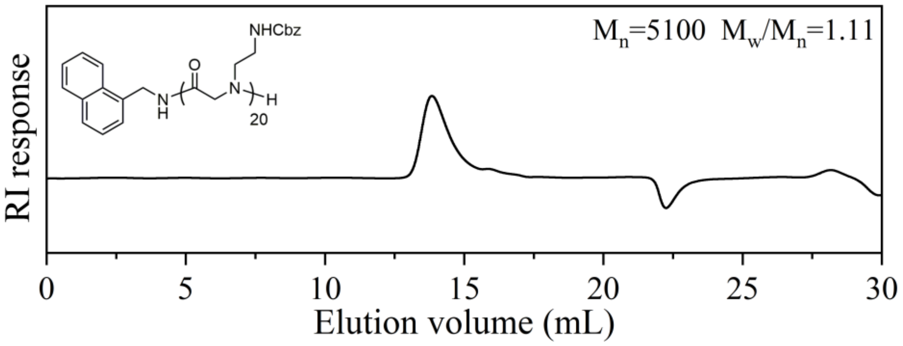

Supplementary Figure 9. GPC trace of N-Cbz protected Polymer 2 using DMF as the mobile phase at a flow rate of 1 mL/min.

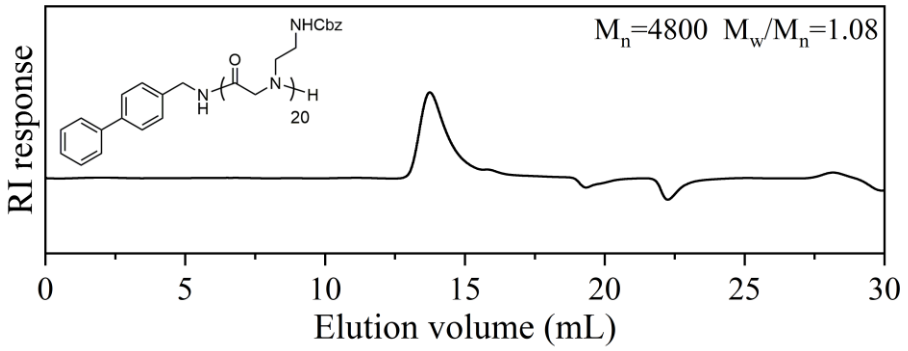

Supplementary Figure 10. GPC trace of N-Cbz protected Polymer 3 using DMF as the mobile phase at a flow rate of 1 mL/min.

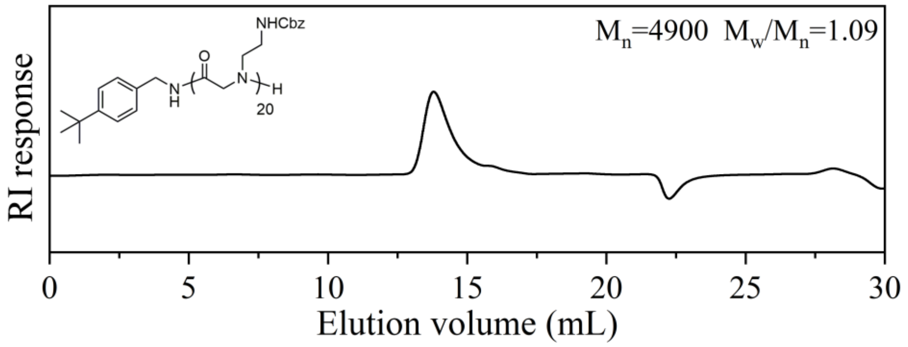

Supplementary Figure 11. GPC trace of N-Cbz protected Polymer 4 using DMF as the mobile phase at a flow rate of 1 mL/min.

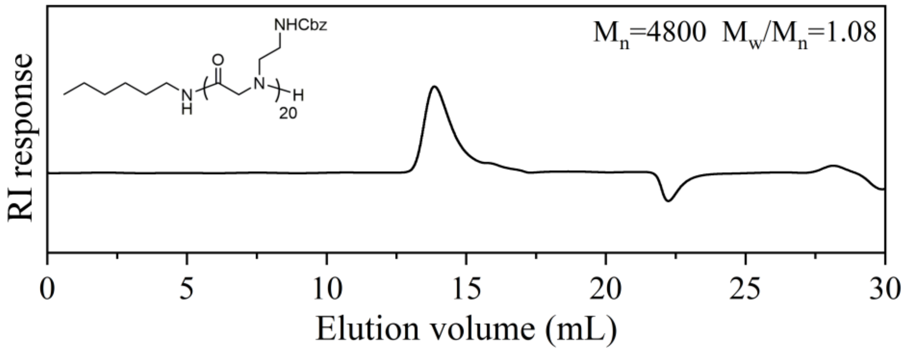

Supplementary Figure 12. GPC trace of N-Cbz protected Polymer 5 using DMF as the mobile phase at a flow rate of 1 mL/min.

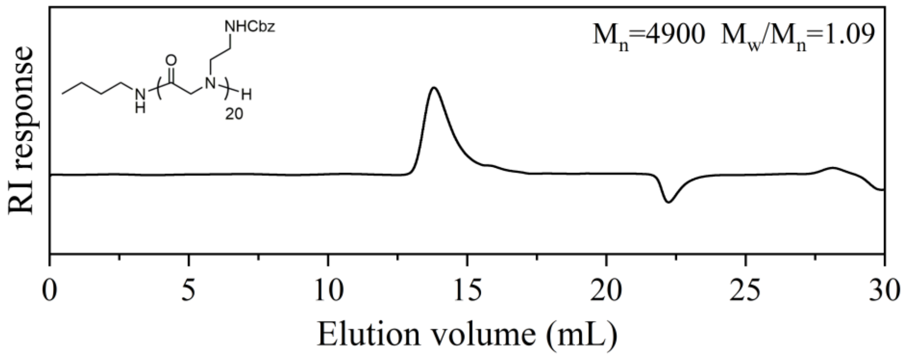

Supplementary Figure 13. GPC trace of N-Cbz protected Polymer 6 using DMF as the mobile phase at a flow rate of 1 mL/min.

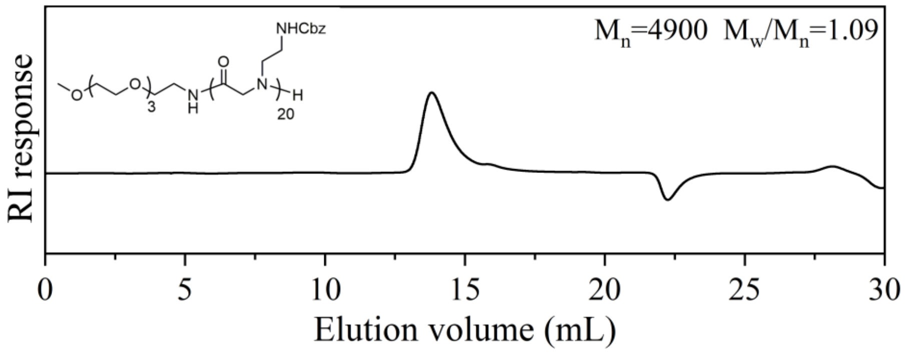

Supplementary Figure 14. GPC trace of N-Cbz protected Polymer 7 using DMF as the mobile phase at a flow rate of 1 mL/min.

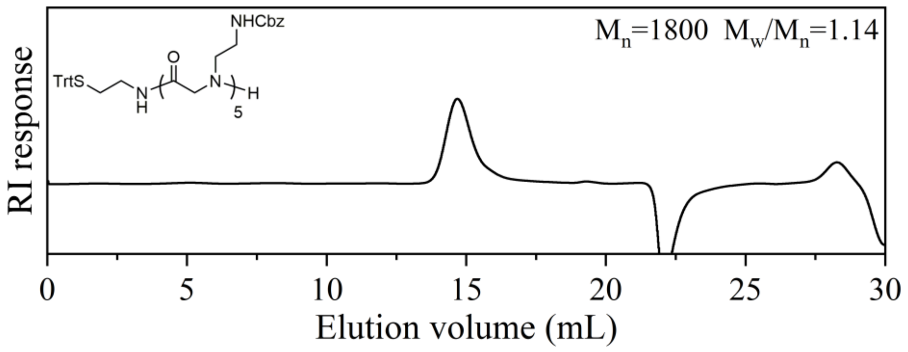

Supplementary Figure 15. GPC trace of N-Cbz protected HS(Naeg)$_5$ using DMF as the mobile phase at a flow rate of 1 mL/min.

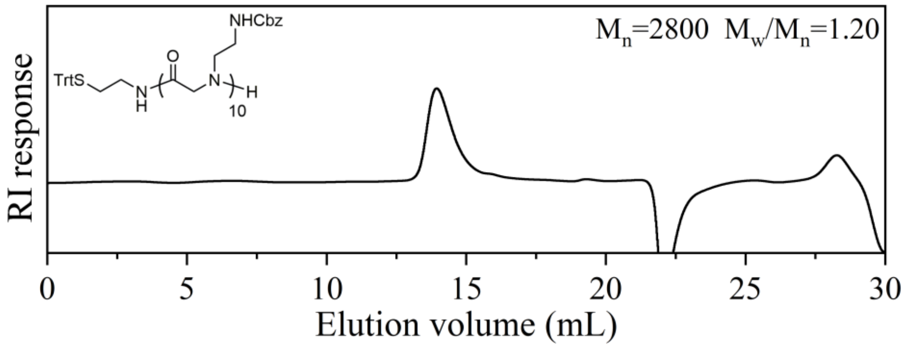

Supplementary Figure 16. GPC trace of N-Cbz protected HS(Naeg)$_{10}$ using DMF as the mobile phase at a flow rate of 1 mL/min.

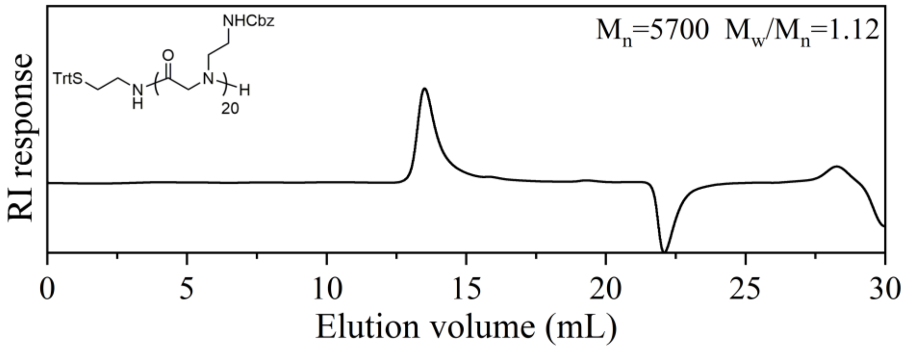

Supplementary Figure 17. GPC trace of N-Cbz protected HS(Naeg)$_{20}$ using DMF as the mobile phase at a flow rate of 1 mL/min.

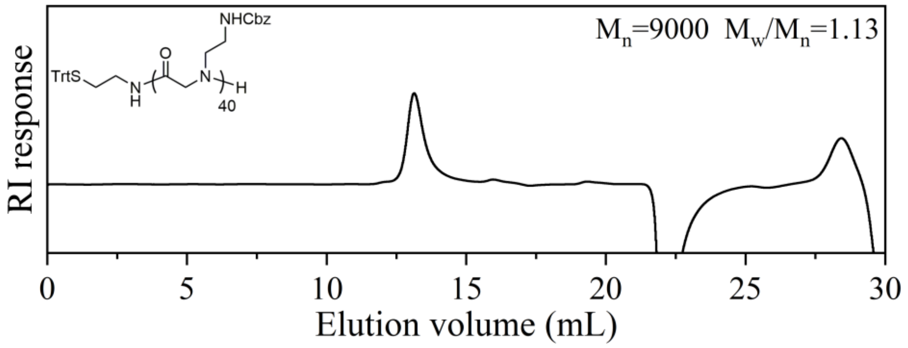

Supplementary Figure 18. GPC trace of N-Cbz protected HS(Naeg)$_{40}$ using DMF as the mobile phase at a flow rate of 1 mL/min.

Deprotection of N-Cbz protected poly-(Naeg)$_n$.

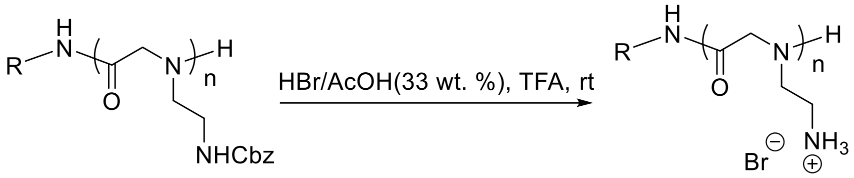

Deprotection of poly-Naeg bearing 2-[(Triphenylmethyl)thio]ethanamine as the terminal group requires addition of Triethylsilane during deprotection.

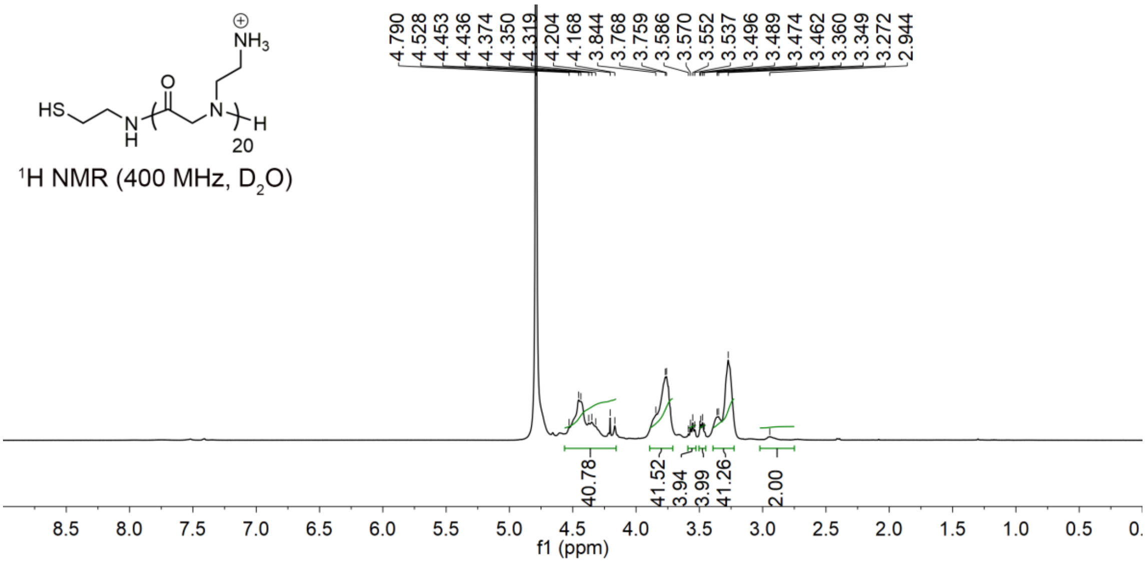

Supplementary Figure 19. ^{1}H NMR spectrum of Polymer 1 in D_2O.

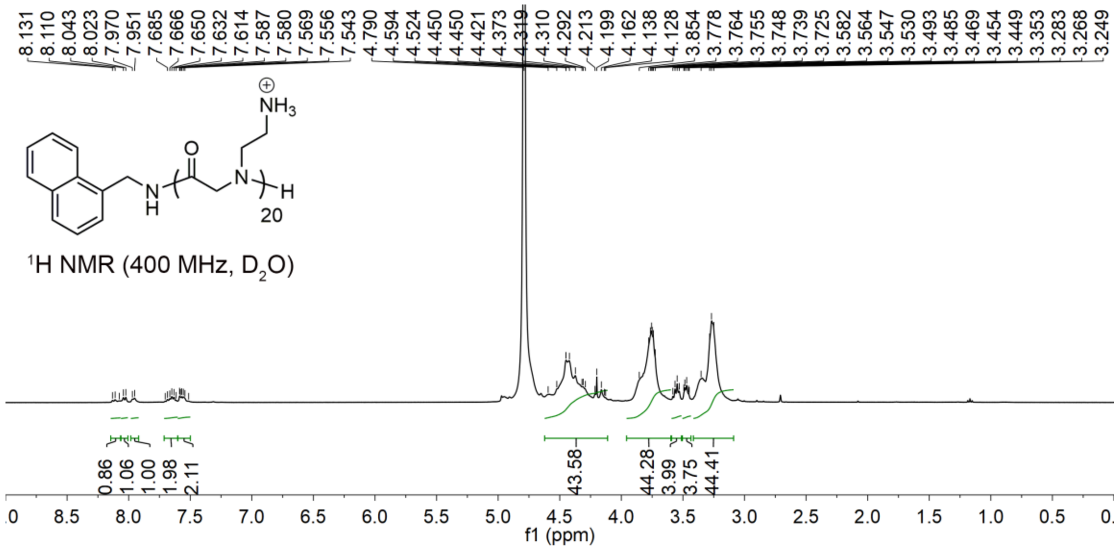

Supplementary Figure 20. ^{1}H NMR spectrum of Polymer 2 in D_2O.

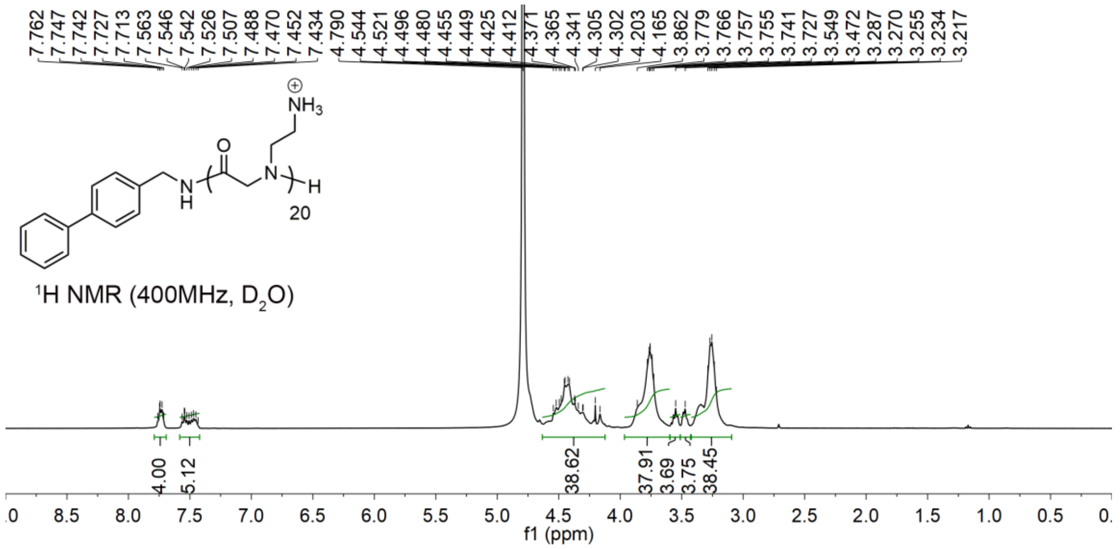

Supplementary Figure 21. ^{1}H NMR spectrum of Polymer 3 in D_2O.

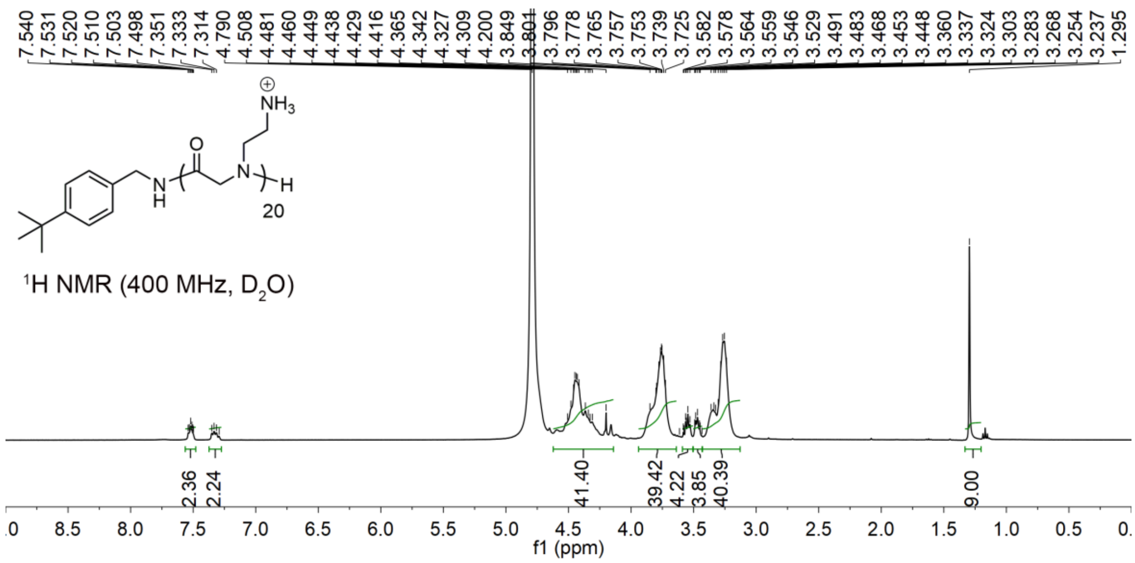

Supplementary Figure 22. ^{1}H NMR spectrum of Polymer 4 in D_2O.

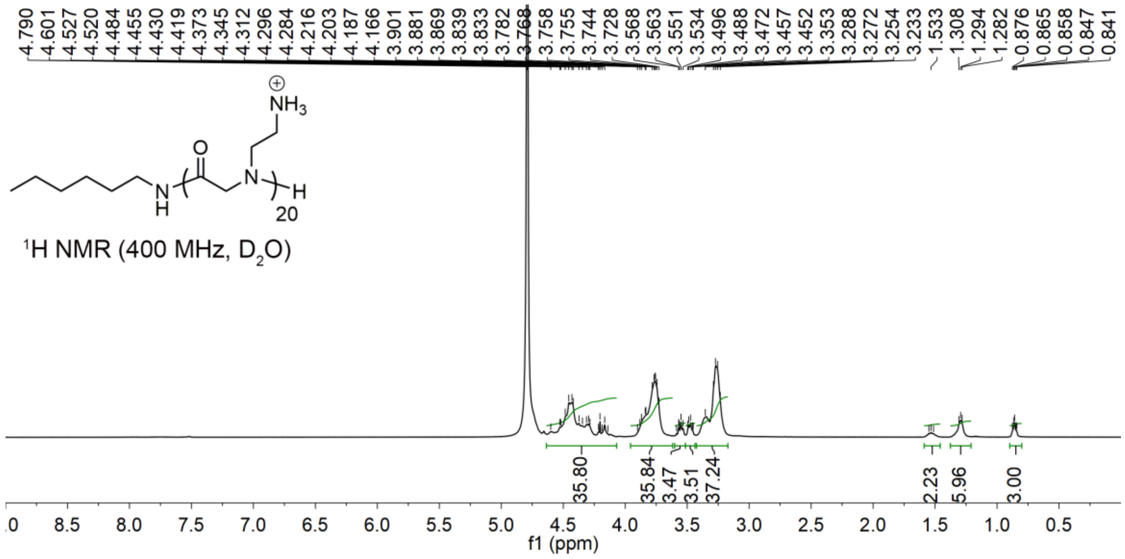

Supplementary Figure 23. ^{1}H NMR spectrum of Polymer 5 in D_2O.

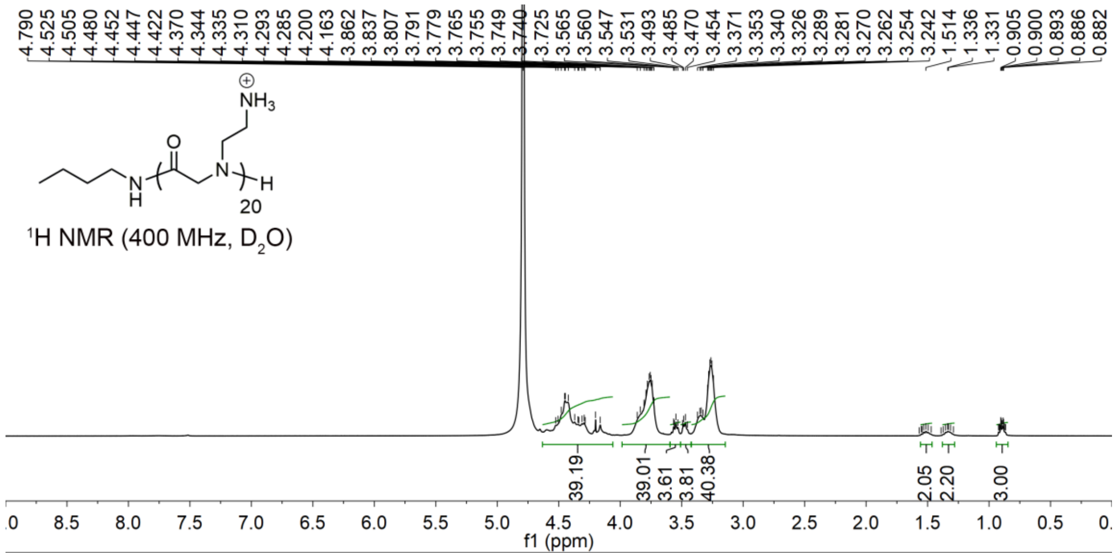

Supplementary Figure 24. ^{1}H NMR spectrum of Polymer 6 in D_2O.

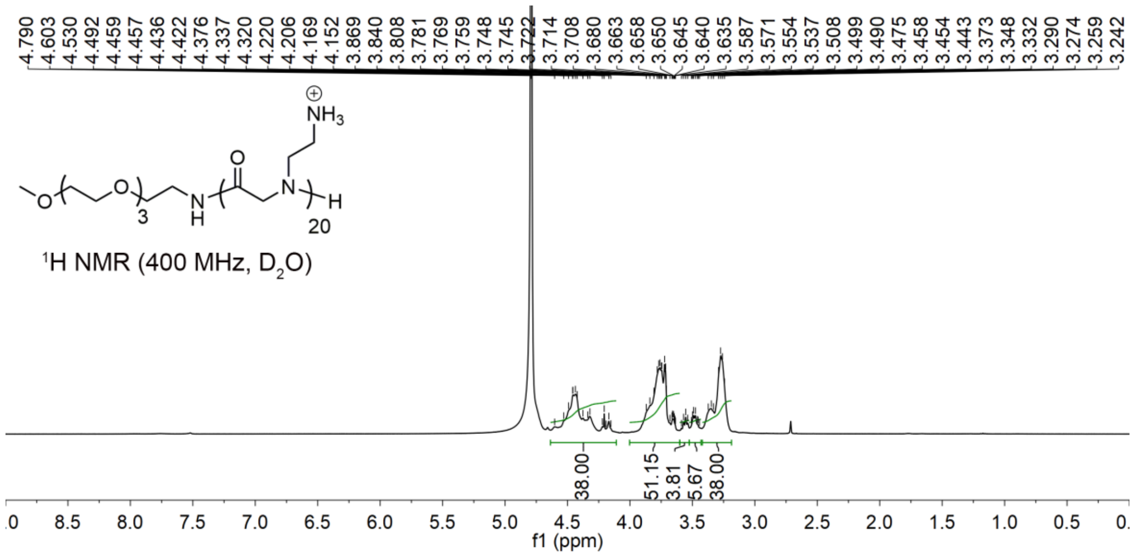

Supplementary Figure 25. ^{1}H NMR spectrum of Polymer 7 in D_2O.

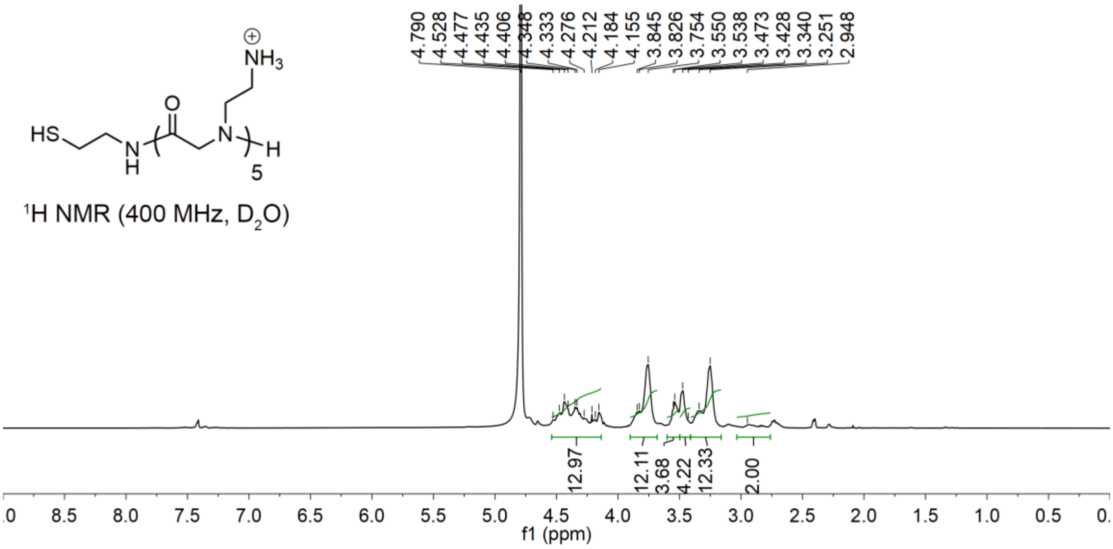

Supplementary Figure 26. ^{1}H NMR spectrum of HS(Naeg)$_5$ in D_2O.

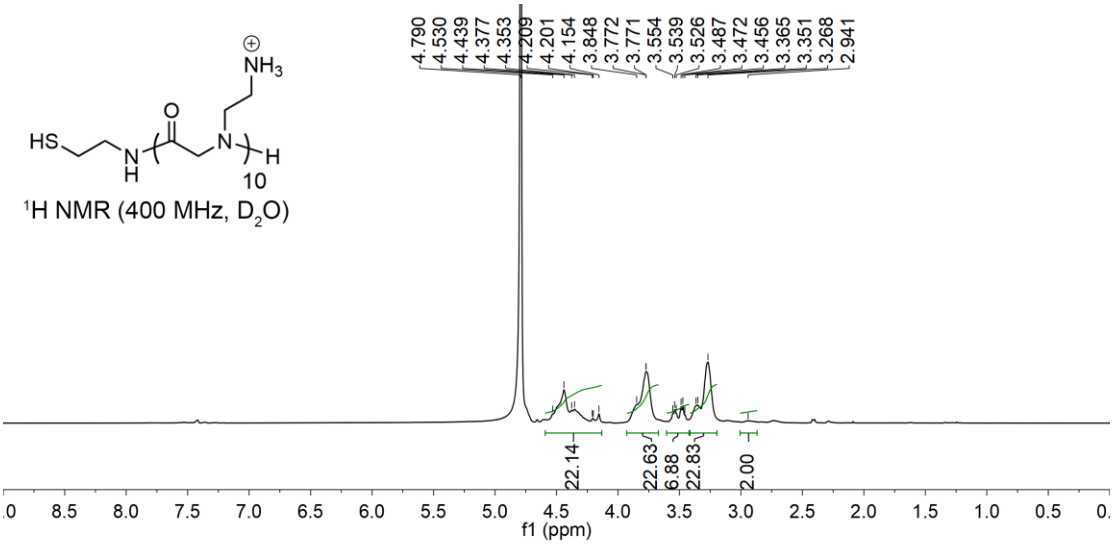

Supplementary Figure 27. ^{1}H NMR spectrum of HS(Naeg)$_{10}$ in D_2O.

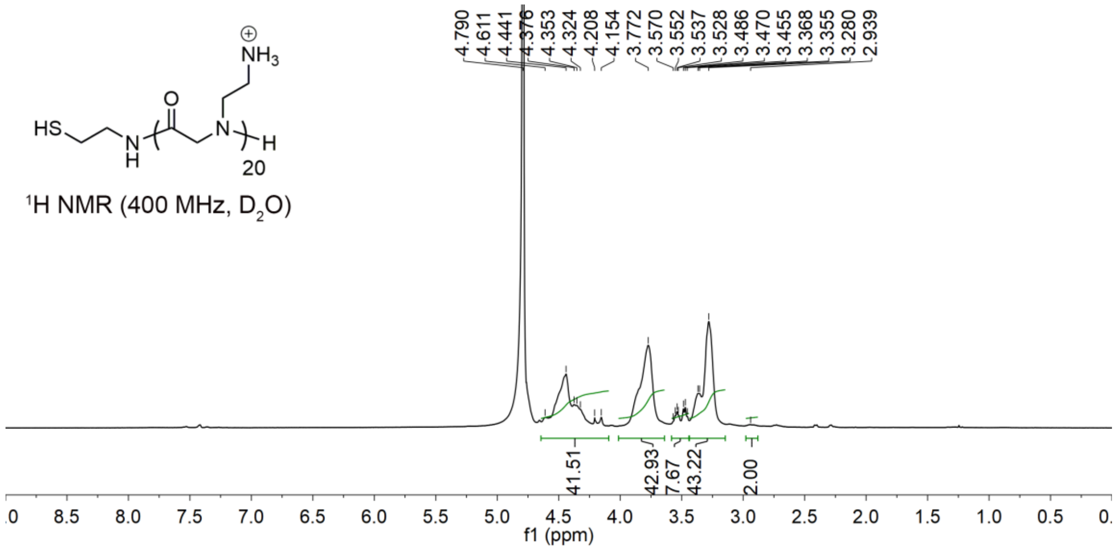

Supplementary Figure 28. **^{1}H NMR spectrum of HS(Naeg)$_{20}$ in D_2O.**

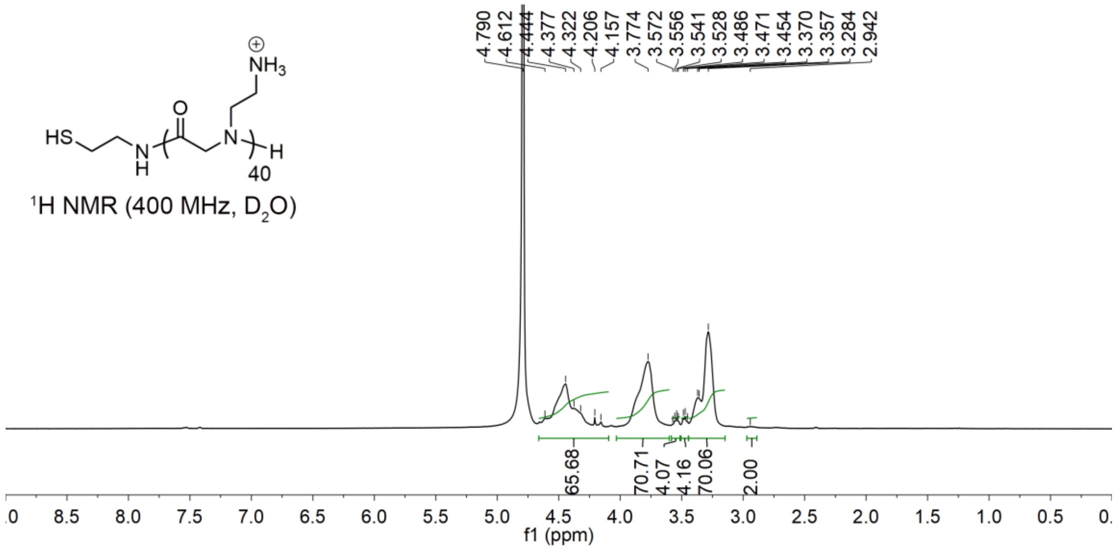

Supplementary Figure 29. **^{1}H NMR spectrum of HS(Naeg)$_{40}$ in D_2O.**

Synthesis of N-(3-aminopropyl)-4-morpholine-1,8-naphthalimide.

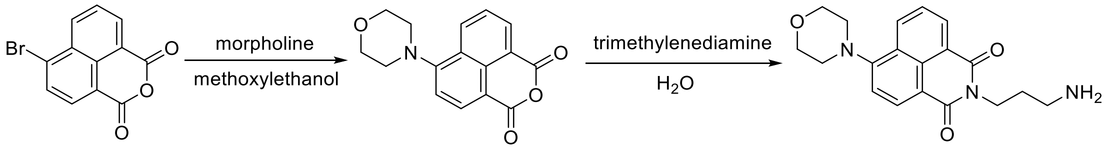

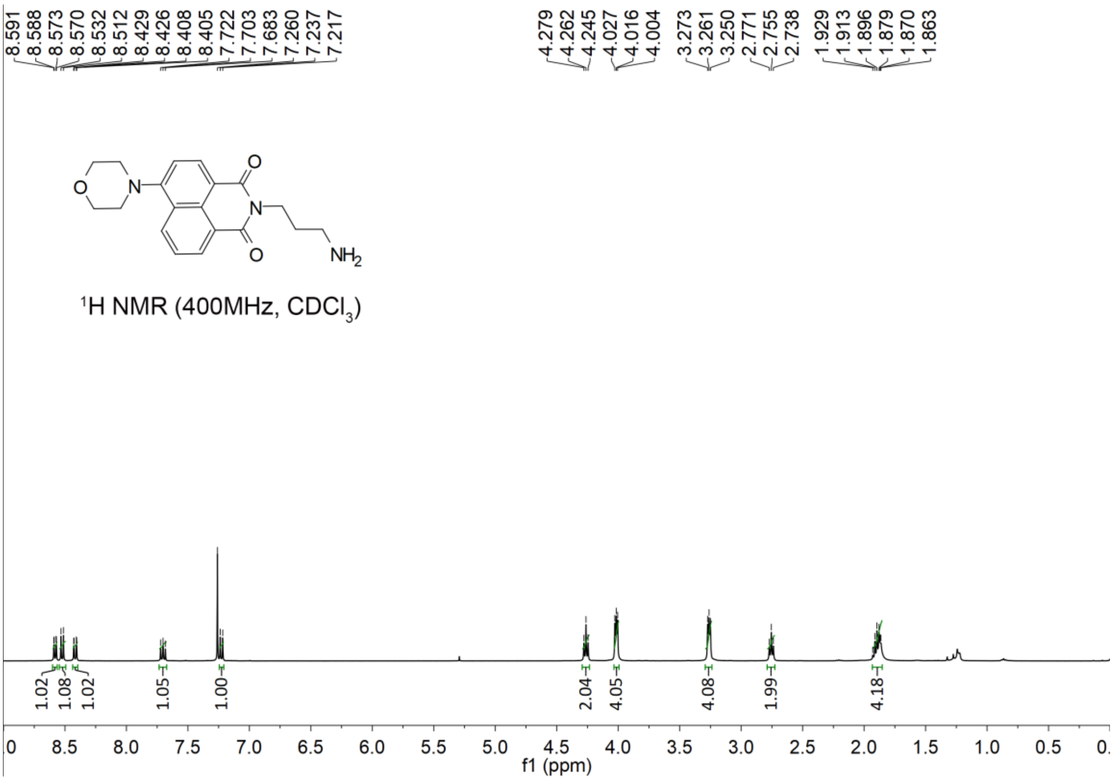

Supplementary Figure 30. ^1H NMR spectrum of dye-NH_2 in $CDCl_3$.

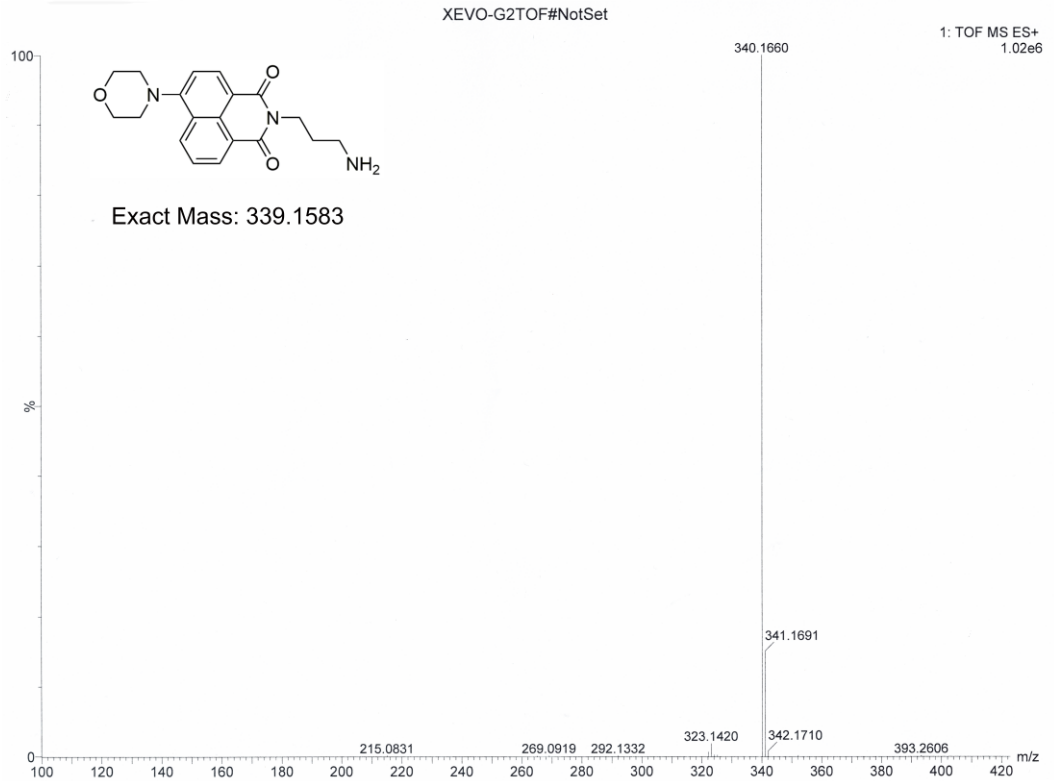

Supplementary Figure 31. HRESI-MS spectrum of dye-NH_2.

Synthesis of Dye-(Naeg)$_{20}$.

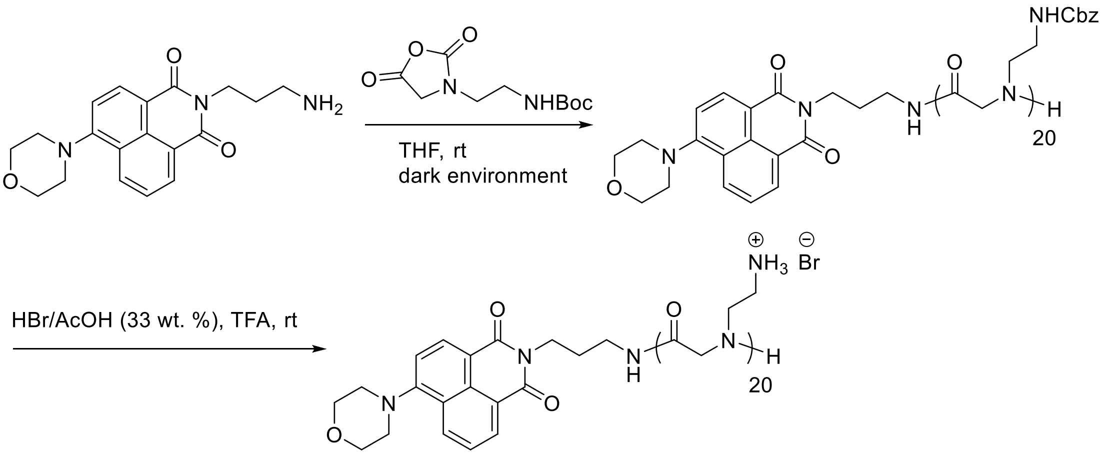

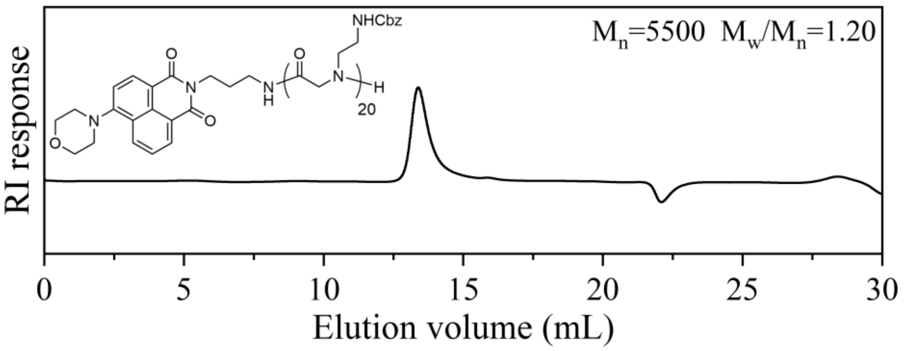

Supplementary Figure 32. GPC trace for N-Cbz protected Dye-(Naeg)$_{20}$ using DMF as the mobile phase at a flow rate of 1 mL/min.

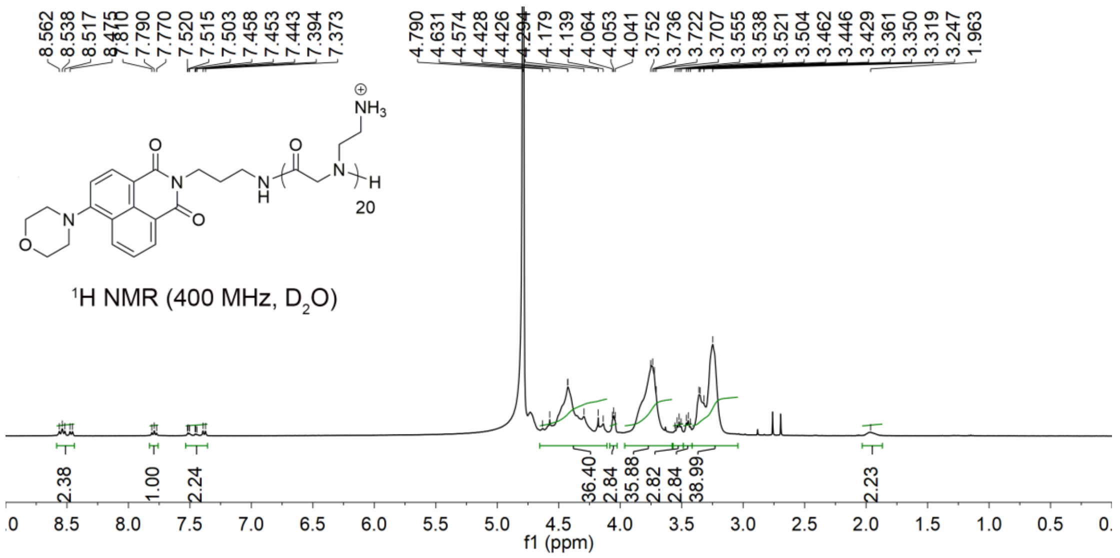

Supplementary Figure 33. ^{1}H NMR spectrum of Dye-(Naeg)$_{20}$ in D_2O.

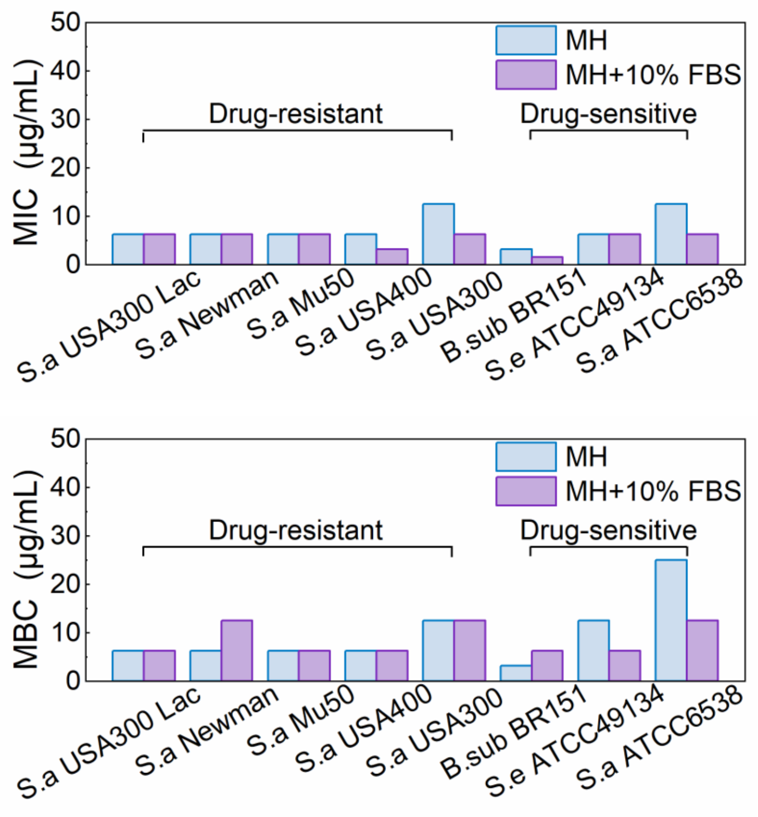

Supplementary Figure 34. The MIC and MBC values of HS(Naeg)$_{20}$ against eight strains of Gram-positive bacteria using MH containing 10% FBS as the medium.

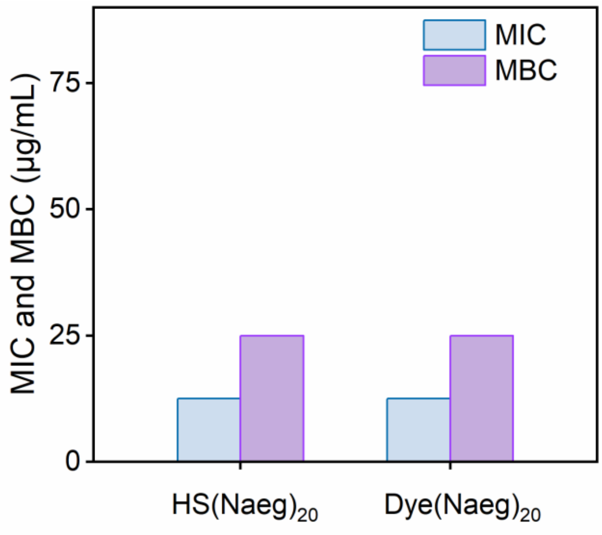

Supplementary Figure 35. MIC and MBC of the Dye-(Naeg)$_{20}$ against *S. aureus* in comparison with HS(Naeg)$_{20}$. The identical antibacterial activity of the Dye-(Naeg)$_{20}$ and HS(Naeg)$_{20}$ supported that morpholino-naphthalimide labelled Poly-Naeg was suitable for antibacterial mechanism study.

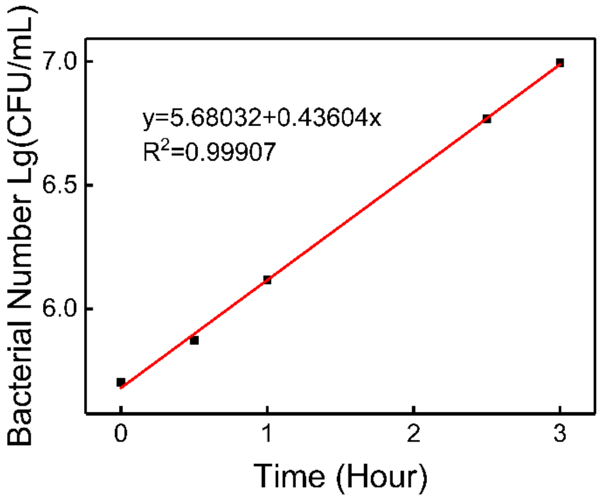

Supplementary Figure 36. Rate of *S. aureus* growth in the presence of HS(Naeg)$_{20}$ at 0.5×MBC.

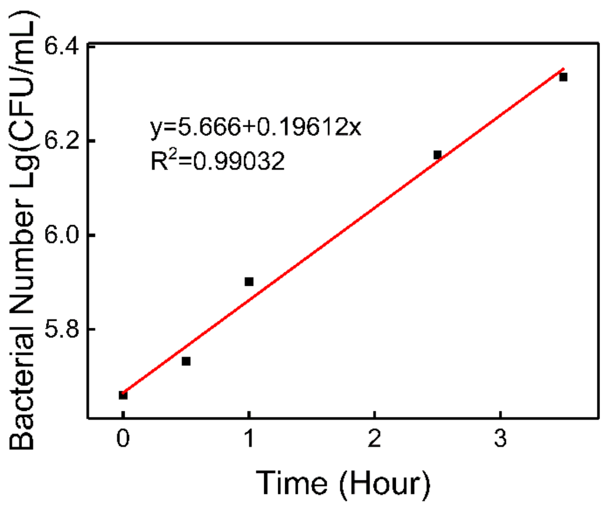

Supplementary Figure 37. Rate of *S. aureus* growth in the presence of norfloxacin at 0.5×MBC.

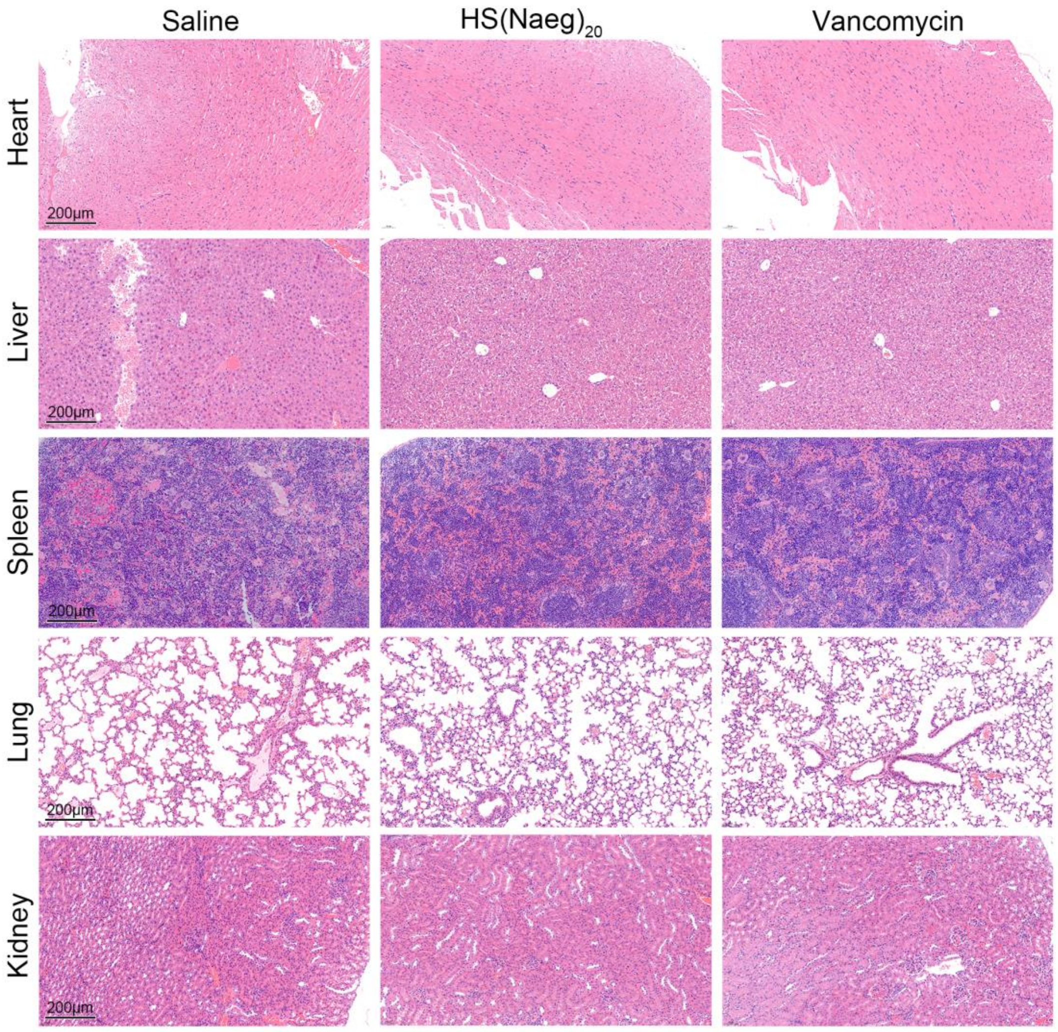

Supplementary Figure 38. Histology analysis of heart, liver, spleen, lung and kidney in saline, vancomycin and HS(Naeg)$_{20}$ treated mice in the mouse peritonitis model. Magnification, ×400.

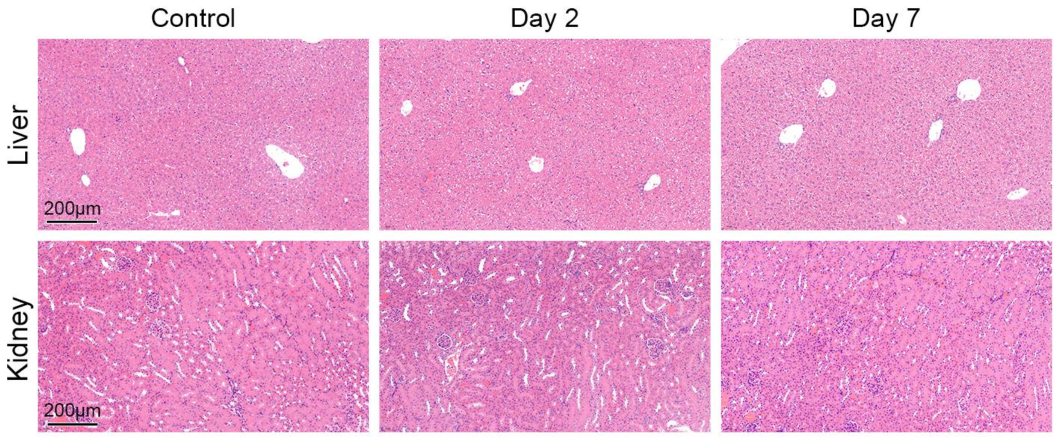

Supplementary Figure 39. ***In vivo*** **toxicity study of HS(Naeg)$_{20}$. Histology analysis of liver and kidney of mice at 2 and 7 days post-treatment with polymer. The untreated mice served as a blank control. Magnification, ×400.**

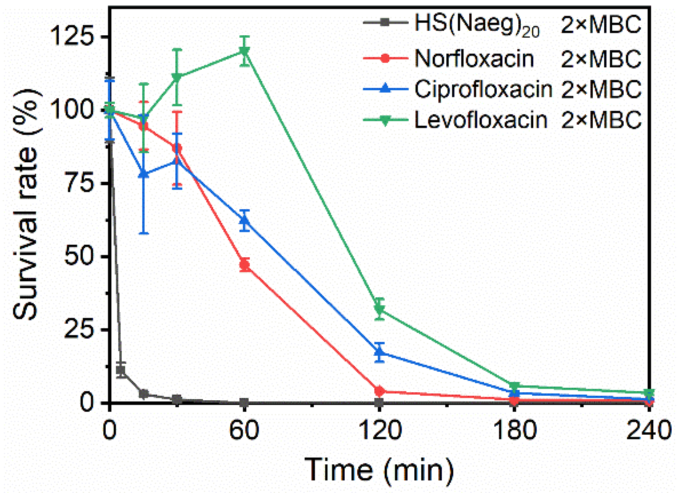

Supplementary Figure 40. Bacterial killing kinetics of HS(Naeg)$_{20}$, norfloxacin, ciprofloxacin and levofloxacin against *S. aureus* ATCC6538 at a concentration of 2×MBC. *n* = 3 per group, data are presented as mean ± SD.

Supplementary Table 1. MIC and MBC values of HS(Naeg)$_{20}$ in the presence of physiologically relevant salts against *S. aureus* USA300 and *S. aureus* USA300 LAC.

strain	MIC/MBC (μg/mL)				
	No salts added	1mM $MgCl_2$	2mM $CaCl_2$	150mM NaCl	200mM NaCl
S. aureus USA300	12.5/12.5	12.5/12.5	12.5/12.5	12.5/12.5	12.5/12.5
S. aureus USA300 LAC	6.25/6.25	6.25/6.25	6.25/6.25	6.25/6.25	6.25/6.25
