# Supplementary material for: Addressing MRSA infection and antibacterial resistance with peptoid polymers
Source: Nat Commun. 2021 Oct 8;12:5898. doi: 10.1038/s41467-021-26221-y (PMC8501045; doi:10.1038/s41467-021-26221-y)
Supplement: Supplementary file 3 — Reporting Summary [file 41467_2021_26221_MOESM3_ESM.pdf]

nature research

Corresponding author(s): Runhui Liu

Last updated by author(s): Sep 9, 2021

Reporting Summary

Nature Research wishes to improve the reproducibility of the work that we publish. This form provides structure for consistency and transparency in reporting. For further information on Nature Research policies, see our Editorial Policies and the Editorial Policy Checklist.

Statistics

For all statistical analyses, confirm that the following items are present in the figure legend, table legend, main text, or Methods section.

n/a	Confirmed	
☐	☒	The exact sample size (*n*) for each experimental group/condition, given as a discrete number and unit of measurement
☐	☒	A statement on whether measurements were taken from distinct samples or whether the same sample was measured repeatedly
☐	☒	The statistical test(s) used AND whether they are one- or two-sided<br>*Only common tests should be described solely by name; describe more complex techniques in the Methods section.*
☒	☐	A description of all covariates tested
☐	☒	A description of any assumptions or corrections, such as tests of normality and adjustment for multiple comparisons
☐	☒	A full description of the statistical parameters including central tendency (e.g. means) or other basic estimates (e.g. regression coefficient) AND variation (e.g. standard deviation) or associated estimates of uncertainty (e.g. confidence intervals)
☐	☒	For null hypothesis testing, the test statistic (e.g. *F*, *t*, *r*) with confidence intervals, effect sizes, degrees of freedom and *P* value noted<br>*Give P values as exact values whenever suitable.*
☒	☐	For Bayesian analysis, information on the choice of priors and Markov chain Monte Carlo settings
☒	☐	For hierarchical and complex designs, identification of the appropriate level for tests and full reporting of outcomes
☒	☐	Estimates of effect sizes (e.g. Cohen's *d*, Pearson's *r*), indicating how they were calculated

Our web collection on statistics for biologists contains articles on many of the points above.

Software and code

Policy information about availability of computer code

Data collection	For data collection we used AVANCE III 400 spectrometer (400 MHz) with TopSpin software (version:3.1), Ascend 600 spectrometer (600 MHz) with TopSpin software (version:4.0.3), Waters XEVO G2 TOF mass spectrometer with MassLynxTM software (version:4.1), Waters GPC instrument equipped with an isocratic HPLC pump (Waters 1515) and a refractive index detector (Waters 2414) with Breeze 2 software (version: 6.20.00.00), Hitachi S-4800 Field Emission Scanning Electron Microscope operated with FE-PC SEM software (version: 3.18), FEI Tecnai Spirit Transmission Electron Microscope with DM software, Microplate reader (Molecular Devices SpectraMax M2 precision), ZEISS LSM 880 with Airyscan with ZEN system (version: 2.3).
Data analysis	Excel (version: 16.0.4266.1001), Origin (version: 9.65.169), MestReNova (version: 11.0.18998), Breeze 2 (version: 2154-C), CaseViewer (version: 2.3.0.99276), ZEN 2.5 (version: 2.5.75.0).

For manuscripts utilizing custom algorithms or software that are central to the research but not yet described in published literature, software must be made available to editors and reviewers. We strongly encourage code deposition in a community repository (e.g. GitHub). See the Nature Research guidelines for submitting code & software for further information.

Data

Policy information about availability of data

All manuscripts must include a data availability statement. This statement should provide the following information, where applicable:

- Accession codes, unique identifiers, or web links for publicly available datasets
- A list of figures that have associated raw data
- A description of any restrictions on data availability

Data that support the findings detailed in this study are available in the Supplementary Information and this article. The source data underlying Figs. 2a, 3a, 3c, 3e, 3f, 4, 5, 6, Table 1, Supplementary Figs. 36, 37, 40 and Supplementary Table 1 are provided in the Source Data file. Any other source data perceived as pertinent are

available, on reasonable request, from the corresponding author.

Field-specific reporting

Please select the one below that is the best fit for your research. If you are not sure, read the appropriate sections before making your selection.

☒ Life sciences ☐ Behavioural & social sciences ☐ Ecological, evolutionary & environmental sciences

For a reference copy of the document with all sections, see nature.com/documents/nr-reporting-summary-flat.pdf

Life sciences study design

All studies must disclose on these points even when the disclosure is negative.

Sample size	No sample size calculation was performed in advance. Samples size are determined according to comparable experiments in previously articles. N=5 mice were used for each arm of in vivo toxicity study. N=7 mice were used for each arm of full-thickness wound model. N=4 mice were used for each arm of keratitis model. N=6 mice were used for each arm of peritonitis model.
Data exclusions	The authors declare no data exclusion.
Replication	Mouse keratitis model and in vivo systemic toxicity study were performed once. All other in vitro and in vivo experiments were reliably repeated at least two times. For the mouse keratitis model and in vivo systemic toxicity study, reproducibility was verified by similar results for individual animal within the group. IACUC approval requires that the minimum possible number of animals be used that yield statistically significant results.
Randomization	Randomization used for in vivo study. For experiments other than those performed in vivo, such as bacteria killing kinetics study , randomized conditions were not necessary.
Blinding	All in vivo experiments were blinded for data processing. For experiments other than those performed in vivo, experiments were not blinded.

Reporting for specific materials, systems and methods

We require information from authors about some types of materials, experimental systems and methods used in many studies. Here, indicate whether each material, system or method listed is relevant to your study. If you are not sure if a list item applies to your research, read the appropriate section before selecting a response.

Materials & experimental systems

n/a	Involved in the study	
☒	☐	Antibodies
☐	☒	Eukaryotic cell lines
☒	☐	Palaeontology and archaeology
☐	☒	Animals and other organisms
☐	☒	Human research participants
☒	☐	Clinical data
☒	☐	Dual use research of concern

Methods

n/a	Involved in the study	
☒	☐	ChIP-seq
☒	☐	Flow cytometry
☒	☐	MRI-based neuroimaging

Eukaryotic cell lines

Policy information about cell lines

Cell line source(s)	NIH 3T3 fibroblast cells (ATCC CRL-1658) were obtained from the Cell Bank of the Chinese Academy of Sciences (Shanghai, China).
Authentication	NIH 3T3 fibroblast cells (ATCC CRL-1658) were not authenticated by us repeatedly.
Mycoplasma contamination	No mycoplasma contamination.
Commonly misidentified lines (See ICLAC register)	No commonly misidentified lines.

Animals and other organisms

Policy information about studies involving animals; ARRIVE guidelines recommended for reporting animal research

Laboratory animals	8 week female BALB/c mice were used for in vivo efficacy study in MRSA full-thickness wound model. 6 week female ICR mice were used for in vivo efficacy study in S. epidermidis and S. haemolyticus full-thickness wound model, in vivo systemic toxicity study and

	acute systemic efficacy study. 8 week male BALB/c mice were used for in vivo efficacy study in keratitis model. Mice were raised in an IVC system at 20-26 °C and 40-70% humidity, with a dark/light cycle of 12 h.
Wild animals	None
Field-collected samples	None
Ethics oversight	All animal procedures were performed in accordance with the Guidelines for Care and Use of Laboratory Animals of the Ninth People's Hospital, Shanghai Jiao Tong University School of Medicine and experiments were approved by the Animal Ethics Committee of the Ninth People's Hospital, Shanghai Jiao Tong University School of Medicine. The laboratory animal usage license number is SYXK-2016-0016, certified by Science and Technology commission of Shanghai Municipality.

Note that full information on the approval of the study protocol must also be provided in the manuscript.

Human research participants

Policy information about studies involving human research participants

Population characteristics	Human blood was collected from male and female donors aged from 18 to 55.
Recruitment	Human blood donors were recruited under the approval of the Ninth People's Hospital, Shanghai Jiao Tong University School of Medicine with informed consent. There is no potential self-selection bias or other biases to impact the results.
Ethics oversight	The Ninth People's Hospital, Shanghai Jiao Tong University School of Medicine.

Note that full information on the approval of the study protocol must also be provided in the manuscript.
